# Assuring Access to Data for Chemical Evaluations

**DOI:** 10.1289/ehp.1206101

**Published:** 2012-12-11

**Authors:** Lynn R. Goldman, Ellen K. Silbergeld

**Affiliations:** 1 School of Public Health and Health Services, The George Washington University, Washington, DC, USA; 2 Department of Environmental Health Sciences, Johns Hopkins Bloomberg School of Public Health, Baltimore, Maryland, USA

**Keywords:** access to information, chemicals, hazardous, pesticides, review, systematic

## Abstract

Background: A database for studies used for U.S. Environmental Protection Agency (EPA) pesticide and chemical reviews would be an excellent resource for increasing transparency and improving systematic assessments of pesticides and chemicals. There is increased demand for disclosure of raw data from studies used by the U.S. EPA in these reviews.

Objectives: Because the Information Quality Act (IQA) of 2001 provides an avenue for request of raw data, we reviewed all IQA requests to the U.S. EPA in 2002–2012 and the U.S. EPA’s responses. We identified other mechanisms to access such data: public access databases, the Freedom of Information Act (FOIA), and reanalysis by a third party.

Discussion: Only two IQA requests to the U.S. EPA were for raw data. Both of these were fulfilled under FOIA, not the IQA. Barriers to the U.S. EPA’s proactive collection of all such data include costs to the U.S. EPA and researchers, significant time burdens for researchers, and major regulatory delays. The U.S. EPA regulatory authority in this area is weak, especially for research conducted in the past, not funded by the U.S. government, and/or conducted abroad. The U.S. EPA is also constrained by industry confidential business information (CBI) claims for regulatory testing data under U.S. chemical and pesticide laws. The National Institutes of Health Clinical Trials database systematically collects statistical data about clinical trials but not raw data; this database may be a model for data from studies of chemicals and pesticides.

Conclusions: A database that registers studies and obtains systematic sets of parameters and results would be more feasible than a system that attempts to make all raw data available proactively. Such a proposal would not obviate rights under the IQA to obtain raw data at a later point.

The U.S. Environmental Protection Agency (EPA) is one among many agencies covered by the Information Quality Act (IQA 2001), an amendment to the Treasury and General Government Appropriations Act for fiscal year 2001 that has been viewed as a mechanism to increase access to such information and to seek corrections if parties think that government agencies have used faulty information and analyses. The Office of Management and Budget (OMB) issued IQA guidelines that apply to all agencies in the Executive Branch: When these agencies provide “influential scientific, financial, or statistical information,” they also “shall include a high degree of transparency about data and methods to facilitate the reproducibility of such information by qualified third parties” (OMB 2002). The law was enacted without debate or hearing. In the absence of an extensive legislative history and because both the IQA and OMB guidelines were silent about whether agency responses were judicially reviewable, some had viewed the act as providing a new avenue for legal challenges of agency decisions across the U.S. government. For example, in 2006 the U.S. Fourth Circuit Court of Appeals ruled that plaintiffs did not have standing to sue the Department of Health and Human Services under Title III of the IQA to compel access to a study conducted by the National Heart, Lung, and Blood Institute (NHLBI) that was used to support action by the Food and Drug Administration (FDA) on dietary salt (Salt Institute *v.* Leavitt 2006). A number of industry groups had petitioned the NHLBI to make the raw data from the study available so that they could do subgroup reanalyses. The court found that the plaintiffs had received no injury from being denied access to the NHLBI data and thus did not have standing. However, the court also noted that the petitioners had a longstanding right to request the raw data from the study using the Freedom of Information Act (FOIA 1966). In its response, the NHLBI noted that it was preparing a public access data set for release, which it later made available (NHLBI 2005). Although this case was resolved under existing FOIA mechanisms, in the wake of this litigation there has been concern that the IQA does not provide outside parties sufficient access to the data for studies that underlie regulatory decisions made by U.S. government agencies. There is increasing interest in improving the methods by which chemical and pesticide hazards and risks are evaluated not only by government but also by independent scientists (Bucher et al. 2011; Woodruff et al. 2011). This interest has spurred increased demand for transparency and disclosure of the data used by the U.S. EPA to make evaluations that support regulatory decisions for chemicals and pesticides. In this context, we examine the role of the IQA in making such data more accessible and suggest alternative approaches.

## Review of Requests for Data

To find out how responsive the U.S. EPA has been to requests for raw data under the IQA, we reviewed 79 requests filed with the U.S. EPA between 2002 and 2012 either to correct or to reconsider the data that the U.S. EPA used in evaluations supporting its regulatory decisions during that period. Under OMB guidance for the IQA (OMB 2002), parties can request that agencies reconsider or correct any information used to support regulatory decisions; usually these requests are made in the form of letters. The U.S. EPA posted these 79 requests on its web site, according to OMB guidelines (U.S. EPA 2012a). Interestingly, only two of these requested raw data.

The first request for raw data was filed in December 2003 by the Perchlorate Study Group, an industry consortium of manufacturers and users of perchlorate (Aerojet, American Pacific Corporation, Kerr-McGee Chemical, and Lockheed Martin). They requested that the U.S. EPA provide raw data from experimental studies (Girard 2003). The U.S. EPA granted this request in September 2004 and provided access to brain images and contractor’s reports (Gilman 2003).

The second case was filed by the Association of Battery Recyclers (ABR) in October 2008 (Steinwurtzel 2008). Now called America’s Battery Recyclers, and formerly called the Secondary Lead Smelters Association, the ABR is a group of auto and industrial battery recyclers, primary lead producers, and users of recycled lead (America’s Battery Recyclers 2012). The ABR requested raw data from a study of lead toxicity (Lanphear et al. 2005) that was among several published studies relied upon by the U.S. EPA in its development of the National Ambient Air Quality Standard (NAAQS) for lead under the Clean Air Act Amendments (1990). Because the ABR and others had taken the U.S. EPA to court to overturn the lead NAAQ rule at the same time, the U.S. EPA opted to postpone consideration of the request under the IQA pending the decision of the court. In its response to the request, the U.S. EPA noted that concerns about the data analysis had been noted in comments during the rule-making process and that the U.S. EPA had commissioned new external peer reviews of the study (U.S. EPA 2012a) in addition to a reanalysis of the data of Lanphear et al. (Rothenberg and Rothenberg 2005). After the lead NAAQS was upheld in July 2010, the ABR again requested that the U.S. EPA provide access to the Lanphear data (Steinwurtzel 2010). Meanwhile, litigation was filed over the delay in providing the data. This litigation was dropped when the U.S. EPA FOIA office worked out an agreement with the Cincinnati Children’s Medical Center to obtain the Lanphear study data (Lanphear BP, personal communication; Pohl *v.* U.S. EPA et al. 2012). U.S. EPA attorneys determined that access to the data was required under the 1998 Shelby Amendment, which makes federally funded research data accessible to the public under FOIA (Treasury and General Government Appropriations Appropriations Act 1998). Thus, as for the request to the NHLBI to provide data concerning the salt study (Salt Institute *v.* Leavitt 2006), the resolution of the request was managed under FOIA.

Because requests for raw data are few and far between, it has not been onerous for the U.S. EPA to provide such data. Existing mechanisms have provided the ability to reanalyze data by *a*) development and availability of a public-access database (with suitable protections for the human subjects involved in such studies); *b*) provision of raw data via FOIA, for cases in which data are in possession of or can be obtained by the agency (e.g., the perchlorate case cited above); and *c*) reanalysis of data by a third party. As an example of the third mechanism, the widely publicized results from the Harvard Six Cities Study (Dockery et al. 1993) were used by the U.S. EPA in 1997 as a basis for developing new standards for fine particulate matter (≤ 2.5 µm in aerodynamic diameter) air pollution (U.S. EPA 1997). Interested parties, mostly from industry, raised questions about study analysis and interpretation. The raw data were not in the possession of the U.S. EPA, and the U.S. EPA could not compel the submission of these data from Harvard University or the funding source, the American Cancer Society. Under pressure from government agencies and industry, Harvard and the American Cancer Society voluntarily requested that the Health Effects Institute (HEI) step in as a third party to supervise a reanalysis of their data. The HEI [a consortium of industry, academic, and government scientists established by the Clean Air Act Amendments (1990)] provided the data for reanalysis by a third party selected by a science advisory committee that included representation from interested parties who had argued for an independent reanalysis, thus providing a process to address the uncertainties about the analysis and interpretation (HEI 2000).

## Discussion

Over time, the U.S. EPA has come to rely increasingly on a large number of scientific studies to complete reviews for a single chemical. This is illustrated by the case of 2,3,7,8-TCDD (2,3,7,8-tetrachlorodibenzo-*p*-dioxin). In its recent assessment of TCDD, the U.S. EPA identified some 2,000 studies directly relevant to its review of dioxin toxicity. From these, the U.S. EPA selected 10 “key” epidemiologic studies and 74 “key” experimental animal studies. Even for this smaller subset of “key” studies, the raw data for each human study and animal experiment are substantial, and most of the data on TCDD were not in the possession of the U.S. EPA (2012b).

There are several mechanisms through which the U.S. EPA might obtain these data. The U.S. EPA could require that investigators submit their raw data to the agency upon completion of their research as a condition of U.S. EPA funding, but this would not completely solve the problem. Most research evaluated by the U.S. EPA for regulatory decision making is not funded by the U.S. EPA. In these cases, the U.S. EPA would have to undertake an extensive collection of raw data from study investigators, which would be costly to the U.S. EPA and burdensome to the research community. Not insignificantly, this would create major delays in rule making. In terms of resource allocation, it is reasonable to ask how much of the U.S. EPA’s budget could be allocated to accomplish this, and where this would rank relative to other priorities, such as increasing the numbers of priority assessments to meet the U.S. EPA’s statutory goals.

In addition to the burden on the U.S. EPA, there would be a significant burden on the scientific community that produces most of the relevant research, and it is very likely that there would be significant pushback from the academic community under the Paperwork Reduction Act (1995). In fact, the Paperwork Reduction Act, which was enacted to reduce the total amount of paperwork handled by the U.S. government, would not allow the U.S. EPA to undertake such a massive data collection without establishing that the burden imposed upon the research community would be justified by the benefits of providing the data.

At the least, scientists would need funding to respond to requests that are generated as a consequence of the use of their studies by the U.S. EPA rather than any action taken by the investigators themselves. Burdened by other responsibilities and unable to fund such activities from grants provided by sources other than the U.S. EPA, scientists are not likely to voluntarily provide the U.S. EPA with raw data from studies conducted months to decades in the past simply because the U.S. EPA has decided to include those studies in their latest assessment.

Moreover, the U.S. EPA would not have clear legal authority to compel the submission of data from industry, federally funded studies conducted prior to the 1998 Shelby Amendment, studies funded by other federal agencies, or studies that are not funded by the U.S. government, including studies from non-U.S. investigators. We therefore conclude that a regulatory approach, in which the U.S. EPA compels the submission of raw data for all studies reviewed for rule making on pesticides and chemicals, would not be tenable. It could in fact have a chilling effect on the engagement of the global scientific community in research relevant to the protection of human health and the environment. Certainly, this is not in the best interests of science-based policy.

In addition, there are other feasibility issues. In the case of older studies, raw data may not exist or may be difficult to access because of storage on outdated media such as tapes. For epidemiologic studies, consideration would need to be given to ethical issues governing studies of human subjects. These include protection of confidentiality and privacy, and prevention of abuse of the data, for example, by marketing companies who may wish to identify patients with particular medical conditions. Clinical-trials investigators have been working for years to develop ways to disclose data from human studies, including mechanisms for placing data behind a barrier to universal access, so that it is accessible only to those who meet conditions of use. In the case of clinical trials, there are studies in which removal of all identifying data negates its scientific value; therefore access to the data would need to be limited to protect privacy (Hrynaszkiewicz et al. 2010). With adequate resources and planning, these obstacles could be anticipated and/or overcome.

In the case of research data concerning chemicals and pesticides, the U.S. EPA also is constrained by legal constructs that have defined regulatory testing of pesticides as “confidential business information” (CBI) and that require the U.S. EPA to redact certain data and obtain affirmations from recipients that they will not give the remaining data to multinational companies that might seek to register the pesticide to market it in other countries (U.S. EPA Office of Pesticide Programs 2010). The U.S. EPA could improve the web access to summaries and analyses of these data, which are publically available but often difficult to find in web searches. This would not be the same as providing access to raw data. We therefore suggest that, in the short run, industry should work with the U.S. EPA to identify approaches to provide more robust data sets for studies that they submit to the U.S. EPA. The U.S. EPA also could invite companies to voluntarily waive CBI claims on tests of pesticides and chemicals. In the long run, we think that Congress should amend the Toxic Substances Control Act (1976) and the Federal Insecticide, Fungicide, and Rodenticide Act (1972) as amended by the Food Quality Protection Act (1996) to ease CBI protections from pesticide and chemical test data.

In an ideal world we would always favor more disclosure over less, but it is not clear how this should be done, or who should pay for it. The HEI, which has an independent governing board and is supported by a consortium of funders including the U.S. EPA and the automobile and petrochemical industries, may be a useful precedent. The HEI requires that data from all HEI-funded studies be made available as expeditiously as possible,

[taking] into consideration the legitimate intellectual interests of the investigator to have the opportunity to benefit from his or her intellectual endeavors and to publish subsequent analyses from the data set (including additional analyses funded by HEI). (HEI 2010)

The HEI attempts to balance the interests of investigators with those of interested parties in cases of “studies of particularly high regulatory importance being used to inform decisions over a short time frame,” and encourages its principal investigators to share the data except in situations where “providing the data would place an undue burden on the investigator” (HEI 2010). For example, in cases when there have been so many requests that it was difficult for the investigators to continue their research, the HEI has assisted investigators with data sharing. In addition, the HEI requires that data requesters provide “reasonable reimbursement for both the direct costs of providing the data, and for the time of the investigator and/or HEI staff to gather, transmit, and explicate the data” (HEI 2010). HEI also “will consider requests from the investigator for a reasonable budget of data archiving funds, to be provided as part of the project budget” (HEI 2010). From this precedent, it seems that proponents of increased access to raw data need to consider not only financial and time burdens on investigators, but also a way to reasonably balance the need for data access with the ability of investigators to realize the fruits of their own intellectual endeavors.

Another useful precedent that could serve as a model for data sharing is the National Institutes of Health (NIH) clinical trials database (ClinicalTrials.gov; NIH 2012). It does not contain “raw data” but rather contains detailed and useful information about clinical-trial study designs and statistics that not only convey results in a standardized fashion but also identify important quality parameters (e.g., drop-out rates). Required by law (Section 113 of the Food and Drug Administration Modernization Act 1997), the clinical trials database was developed by the NIH with input from the FDA and the National Library of Medicine (NLM). Currently, many medical journals require that trials be registered in ClinicalTrials.gov prior to their publication; as of 3 December 2012, 136,605 studies in 182 countries were registered. Although many researchers are now are calling for access to raw data for all clinical trials (Gotzsche 2011), the ClinicalTrials.gov database has greatly increased access to information about drug efficacy trials and drug safety, and the development of such a database for studies of chemicals and pesticides would be a major step toward increasing the transparency of the U.S. EPA’s evaluations and making data more accessible to third parties.

## Conclusions

At present, there does not seem to be a large demand for raw data related to U.S. EPA decision making; however, this may change as formal evidentiary reviews of environmental health research become increasingly common (Maull et al. 2012). Compared with clinical trials, the acquisition of raw data for chemicals and pesticides would be much more complex, in part because it would require a framework that can accommodate data from numerous types of studies: observational and experimental, animal, human, *in vitro*, and high throughput screening studies.

For human epidemiologic studies, clear and complete documentation would need to be provided for interpretation of the variables collected in such studies. This is no simple task given, for example, *a*) the wide range of possible study designs and the intricacies of design of questionnaires and subsequent coding and transformation of variables; *b*) environmental and biomarker sample-collection procedures, chain-of-custody and sample processing and storage, laboratory analyses, data analysis, and coding; and *c*) imputation of missing variables or laboratory nondetects. Although it is a standard practice to carefully document all of these details, there is currently no generally agreed-on manner in which to upload such data into an electronic database. There is a risk that people who were not involved in data collection can misunderstand these details and thus obtain erroneous results. Some effort would be required to develop a standardized system for reporting this kind of information. For experimental animal studies, there should be parameters related to quality assessment (e.g., blinding of investigators, randomization, housing and care of animals).

If the U.S. EPA chose this path, the first step might be to develop a framework similar to ClinicalTrials.gov that would capture statistics and other parameters but would not necessarily require uploading raw data. With adequate funding, involvement of the NLM might provide more sophisticated informatics expertise to make the data more usable, and the NLM or the National Toxicology Program (NTP) could perhaps provide a “home” for the data. The U.S. EPA and other environmental agencies could also require (or request) that investigators register their studies with the database, and journals could require registration as a condition of publication (as some journals currently do for results of clinical trials) or suggest that it be done. Given resource limitations, especially for investigators in developing countries, this step might be difficult for many investigators compared with researchers who perform clinical trials.

A system that provides raw data might be possible if the U.S. EPA could pilot the development of a system that could handle raw data using data already in its possession [e.g., results of its intramural research, results of U.S. EPA-funded extramural research (where available), and any raw data that it has requested from investigators in support of risk-assessment activities]. Other federal agencies, such as the NTP and the National Institute for Occupational Safety and Health could contribute as well. The NTP already publishes all of its data and methods in its reports; however, it does not publish raw data or studies with nonstandard protocols online. In any case, busy investigators may oppose this not only because of the effort and resources required but also because they would be relinquishing exclusive access to their own raw data (and therefore the risk of being “scooped”) for the possibility of future requests for reanalysis. Even in cases where investigators contemplate no further data analyses, they may have concerns about the effort to respond to questions about repeat analyses. In any case, additional resources would be required, and this is not a time of plenty for research in the United States or anywhere else. In short, as in all of life, there is no free lunch. We already have mechanisms for disclosure of data used by the U.S. EPA in decision making and even for obtaining raw data. It is doubtful that we can afford the luxury of having this information available for release prior to any request, and it is uncertain who should be responsible for the cost and effort required to provide it.

We conclude that, as is the case for clinical trials, a registry for studies that could handle a wide variety of methodologies and methods of analysis and provide a more complete and standardized presentation of statistical results and other parameters than is possible in the peer-reviewed literature would be a tremendous resource to society for increasing transparency and improving assessments of pesticides and chemicals. However, at present, there is no evidence that there is a net social benefit to requiring collection of and access to raw data for all studies utilized by the U.S. EPA prior to requests for such data from interested parties. As a first step the U.S. EPA, NTP, and NLM should begin to generate discussions among agencies and with interested outside parties, including academic researchers and the regulated industry, on the possible creation of a reporting system for environmental health studies of chemicals and pesticides that would systematically collect results and data about studies—but not raw data.
